# Movement-Related Cortical Potential Amplitude Reduction after Cycling Exercise Relates to the Extent of Neuromuscular Fatigue

**DOI:** 10.3389/fnhum.2016.00257

**Published:** 2016-06-01

**Authors:** Jérôme Nicolas Spring, Nicolas Place, Fabio Borrani, Bengt Kayser, Jérôme Barral

**Affiliations:** ^1^Institute of Sport Sciences, Faculty of Social and Political Sciences, University of LausanneLausanne, Switzerland; ^2^Institute of Sport Sciences and Department of Physiology, Faculty of Biology and Medicine, University of LausanneLausanne, Switzerland; ^3^Institute of Sport Sciences, Faculty of Biology and Medicine, University of LausanneLausanne, Switzerland

**Keywords:** EEG, fatigue, *Bereitschaftspotential*, peripheral nerve stimulation, maximal voluntary contraction

## Abstract

Exercise-induced fatigue affects the motor control and the ability to generate a given force or power. Surface electroencephalography allows researchers to investigate movement-related cortical potentials (MRCP), which reflect preparatory brain activity 1.5 s before movement onset. Although the MRCP amplitude appears to increase after repetitive single-joint contractions, the effects of large-muscle group dynamic exercise on such pre-motor potential remain to be described. Sixteen volunteers exercised 30 min at 60% of the maximal aerobic power on a cycle ergometer, followed by a 10-km all-out time trial. Before and after each of these tasks, knee extensor neuromuscular function was investigated using maximal voluntary contractions (MVC) combined with electrical stimulations of the femoral nerve. MRCP was recorded during 60 knee extensions after each neuromuscular sequence. The exercise resulted in a significant decrease in the knee extensor MVC force after the 30-min exercise (−10 ± 8%) and the time trial (−21 ± 9%). The voluntary activation level (VAL; −6 ± 8 and −12 ± 10%), peak twitch (Pt; −21 ± 16 and −32 ± 17%), and paired stimuli (P100 Hz; −7 ± 11 and −12 ± 13%) were also significantly reduced after the 30-min exercise and the time trial. The first exercise was followed by a decrease in the MRCP, mainly above the mean activity measured at electrodes FC1-FC2, whereas the reduction observed after the time trial was related to the FC1-FC2 and C2 electrodes. After both exercises, the reduction in the late MRCP component above FC1-FC2 was significantly correlated with the reduction in P100 Hz (*r* = 0.61), and the reduction in the same component above C2 was significantly correlated with the reduction in VAL (*r* = 0.64). In conclusion, large-muscle group exercise induced a reduction in pre-motor potential, which was related to muscle alterations and resulted in the inability to produce a maximal voluntary contraction.

## Introduction

Prolonged exercise increases the difficulty to perform voluntary motor actions by altering motor control or the capacity to sustain an ongoing effort or to generate maximal force or power (Allen et al., 1995; Jaric et al., 1997, 1999; Gandevia, 2001; Bottas et al., 2004) in other words, fatigue develops. Exercise-induced muscle fatigue can be defined as a reduction in the maximal voluntary contraction force (MVC; Gandevia, 2001) and can be related to alterations occurring at different sites along the motor pathway, from the cortex to the muscle fiber. It is typical to distinguish between the central factors located before the neuromuscular junction (central fatigue), which refers to the neural activity that drives the muscle, and the peripheral factors located after the motor plate at the muscle level (peripheral fatigue).

Standardized investigative methods for examining neuromuscular function, such as peripheral nerve stimulation or transcranial magnetic stimulation, have been extensively used to explore the complex relationship between exercise and fatigue (Gandevia, 2001; Taylor et al., 2006; Barry and Enoka, 2007). Peripheral nerve stimulation provides relevant information about muscular properties such as excitation-contraction coupling. This method also allows for the quantification of suboptimal motor drive for force production by the twitch interpolation technique (Merton, 1954). However, peripheral nerve stimulation alone remains ineffective for differentiating between supraspinal and spinal adaptations. Transcranial magnetic stimulation is an alternative stimulation method that consists in applying an electromagnetic field above the motor cortex and/or the cervicomedullary junction, to evoke motor evoked potentials, and as such transcranial magnetic stimulation provides information about changes in corticospinal excitability/inhibition (Goodall et al., 2012). By combining transcranial magnetic stimulation and peripheral nerve stimulation, it is possible to assess the neuromuscular pathway and to gain insight into potential site(s) of impairment during or following exercise (Gruet et al., 2014).

Because any voluntary physical effort begins and ends in the brain (Kayser, 2003), voluntary contraction is not limited by the motor command *per se* but also by processes upstream from the motor cortex that might limit motor drive and thus contribute to central fatigue (Gruet et al., 2013). Indeed, during voluntary movement, cortical and subcortical regions are involved in the final motor output (Ball et al., 1999; Shibasaki, 2012; Tanaka and Watanabe, 2012). For example, peripheral afferents send projections to the cingulate anterior cortex, the premotor area, the lateral prefrontal cortex and the orbitofrontal cortex (Liu et al., 2002; Liu, 2003; Hilty et al., 2011a; Robertson and Marino, 2016) and thereby participate in modulating motivational and executive processes. Hence, the brain integrates the internal state and the perceptual information to finally modulate the motor output.

Electroencephalography (EEG) appears to be a relevant method for investigating exercise-induced changes in brain activity, particularly because it reflects the spontaneous and immediate activity of neural networks from a wide range of brain systems. During voluntary muscle contraction tasks, EEG allows for investigation of the spontaneous cortical activity related to movement production. The movement-related cortical potential (MRCP) is an event-related potential locked to the onset of movement (Shibasaki and Hallett, 2006). It reflects the preparatory brain activity, taking into account the time factor. First described by Kornhuber and Deecke; (1965a, b) as the *Bereitschaftspotential*, this EEG pattern is characterized by a slow negative shift, starting ~2 s before movement, and reflects neural processes involved in preparing the motor command. The MRCP is composed of two main components, distinguished by their change in slopes occurring ~500 ms before movement onset (Deecke, 1996; Shibasaki and Hallett, 2006). The first component (BP: Bereitschaftspotential) has a moderate steepness and is bilaterally distributed at the fronto-central midline above the supplementary motor area (SMA). BP occurs between 1500 and 500 ms before movement onset. The second component (NS′: negative slope) is contralateral dominant, more pronounced above the primary motor cortex (M1) and occurs between 500 ms before movement onset and movement onset. Within NS′, the motor potential (MP) is observed at movement onset and corresponds to the MRCP peak amplitude.

Performing voluntary movement implies dynamic processes that involve multiple areas within the brain, likely with overlapping activities. At present, there is a divergence in opinion regarding whether the MRCP components reflect different processes (Jahanshahi and Hallett, 2012). Nevertheless, the identification of the generators of MRCP and their related intracortical connections suggest different stages in the movement-generating procedure. The main generator of MRCP is not limited to the primary motor cortex; the pre-SMA, SMA, and cingulate cortex have a major contribution (Jahanshahi and Hallett, 2012). The activity of additional subcortical structures, such as the thalamus, the caudate, the putamen, and the pallidum also participate in scalp surface-recorded activity and cannot be excluded. By manipulating motor preparation with specific tasks, neuroimaging studies have shown increased activity in the SMA in self-generated movement, compared with movements directed by external cues (Deiber et al., 1991, 1996; Jenkins et al., 2000). Some evidence supports sequential activation within this neural network, in which the SMA has a driving effect on M1 (Herz et al., 2012). Thus, we can assume that the early MRCP generated above the SMA represents the cognitive process related to the decision to perform a movement and the preparation, whereas the late MRCP recorded above the primary motor cortex is more likely to represent the motor part of movement production (Arai et al., 2012; Hoffstaedter et al., 2013). Although the functional distinction between components is still unclear, their segmentation helps to differentiate the processes related to movement planning from motor execution.

The MRCP is obtained by repeated single-joint contractions protocols. Some studies have reported that repetitions of the same movement are accompanied by an increase in MRCP amplitude (Johnston et al., 2001; Schillings et al., 2006; Morree et al., 2012), which has been interpreted as a way to compensate for peripheral fatigue, whereas others have reported no modifications (Siemionow et al., 2004; Liu et al., 2005). Schillings et al. (2006) observed an increase in MRCP after handgrip contractions, but did not find any correlation between MRCP modulations and the reduction in force or with any peripheral fatigue parameters. The authors postulated that the increase in the motor cortical activity compensated for a reduction in central efficiency. In their study, Freude and Ullsperger (1987) asked participants to perform self-paced contractions for 30 min (i.e., 150–250 contractions) at 20, 50, and 80% of their MVC. MRCP increased when fatigue developed during exercise at 20 and 80% of MVC but decreased when the contractions were performed in the absence of fatigue at 50% of MVC. The increase in MRCP amplitude was interpreted differently according to the intensity of effort. At 80% of MVC, the results suggested an increase in cortical activation to compensate for peripheral fatigue, whereas at 20% of MVC, they indicated the high degree of concentration and attention required to properly perform the task. Conversely, at 50% of MVC, the reduction in MRCP was interpreted as a decrease in intentional involvement because of task monotony. More recently, Berchicci et al. (2013) investigated simultaneously MRCP, perception of effort, muscle twitch force and EMG activity. Eighteen subjects performed four blocks of isometric knee extension at 40% of MVC (240 2 s long contractions). After averaging the early (block 1–2) and late blocks (block 3–4), the authors performed a cluster analysis to create two groups based on the rating of perceived exertion (RPE) and the peripheral fatigue (muscle twitch force loss). MRCP increased in the group with higher RPE and in the group with greater peripheral fatigue. They also observed higher positive activity in the prefrontal cortex in the group with greater RPE. According to the authors, the protocol used required high cognitive effort to properly perform the task, which could explain the frontal positivity. In summary, MRCP modulations appear to be related to global exercise-induced fatigue, not only to peripheral fatigue. Some factors might influence the modulations of the pre-motor potential, such as the cognitive load and the perceived effort (Freude and Ullsperger, 1987; Slobounov et al., 2004).

The present study is the first to investigate the effects of an acute endurance exercise on a motor task (voluntary knee extensions) by combining pre-motor brain activity and neuromuscular measurements. Our strategy was to use a specific fatigue-generating procedure, different from the task used to quantify MRCP changes, to avoid bias from additional and unwanted mental weariness. We asked our subjects to perform a large-muscle-group exercise (cycling) and to participate in a specific MRCP task (repeated knee extension; before and after cycling) to quantify the changes induced by the fatiguing exercise. The aim of the study as two-fold: (1) to assess the effect of cycling exercise intensity (heavy and severe) on MRCP modulations and (2) to relate the exercise-induced MRCP modulations to the extent of central and peripheral fatigue. We hypothesized that large-muscle-group exercise would induce neuromuscular fatigue and an increase in MRCP amplitude above the premotor and motor area.

## Materials and methods

### Participants

Twenty well-trained male athletes were enrolled in the study after having been informed of the experimental procedure. All the participants completed the Baecke questionnaire to ensure that they were physically active (Baecke et al., 1982). The protocol was approved by the local ethics committee (CERVD: protocol 153/14) and was in agreement with the Declaration of Helsinki. Each subject provided written consent before participation.

### Experimental protocol

The volunteers visited the laboratory on two occasions, for the pre-participation session and for the experimental session. During the pre-participation session, preliminary medical screening confirmed that participants were in good health and had no disorders that could interfere with the experimental procedure. Upon inclusion, they performed a maximal ramp exercise protocol on a cycle ergometer (Lode Excalibur Sport, Groningen, the Netherlands) to measure the first ventilatory threshold (*SV*_1_), peak oxygen consumption ($\overset{\cdot }{V}$*O*_2_ *peak)*, and maximal aerobic power (MAP). The participants warmed up for 6 min at 60 W, after which power was incremented by 30 W/min until voluntary exhaustion. Maximality was considered to have been reached when at least three of the following criteria were met: $\overset{\cdot }{V}$*O*_2_ plateau; respiratory quotient (QR) >1.1; maximal heart rate (HR_max_) >90% of theoretical HR_max_ (i.e., 220-age); or a pedaling rate below 60 rotations per minute despite strong verbal encouragement.

The experimental session occurred within 4 weeks after the pre-participation session. The volunteers were instructed to maintain their usual diet and to avoid severe exercise the day before. They had to avoid alcohol and caffeine consumption over the 12 h preceding the session. The last meal had to be taken at least 2.5 h before the beginning of the test, and water was provided *ad libitum* during the experimental session.

**Figure 1:**
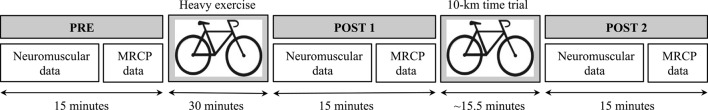
**Experimental session design and timeline**. PRE, pre-exercise condition; POST1, post-heavy exercise condition; POST2, post-time trial condition; MRCP, movement-related cortical potential.

The protocol consisted of heavy exercise, followed 15 min later by severe exercise (Figure 1). The heavy exercise consisted in pedaling at a freely chosen cadence on a cycle ergometer (Lode Excalibur Sport, Groningen, the Netherlands) for 30 min at an intensity of ~60% of MAP. The severe exercise was a 10-km all-out time trial (TT) performed on a road bike with the rear wheel mounted on a home trainer (CycleOps, Madison, USA). The objective was to complete the distance as fast as possible, with the distance displayed on a bike computer fixed to the handlebar. The resistance of the roller increased automatically with the force exerted by the cyclist to reproduce field-like sensations. A fan was placed in front of the subject to avoid excessive sweating, and the wind speed was adjusted upon request. A power meter in the rear hub (PowerTap, CycloOps, Madison, USA) allowed the power output to be recorded. Perceived exertion was assessed with the 6–20 Borg scale at the end of the heavy exercise and the TT. Knee extensor neuromuscular function was investigated through the quantification of several parameters. The MVC represents the maximum force that a subject could produce in the isometric knee exercise. The voluntary activation level (VAL) was chosen as an index of central fatigue and is believed to reflect the ability of the motor cortex to drive muscle. The M-wave was recorded to explore neuromuscular transmission/propagation. The paired stimuli force at 100 Hz (P100 Hz) and the muscle twitch force (Pt) reflected the muscle properties and were both chosen as indices of peripheral fatigue. Neuromuscular data were collected before the fatiguing task (PRE), immediately after the 30-min exercise (POST1) and immediately after the 10-km TT (POST2). The MRCP data were recorded after each neuromuscular assessment for these three time points.

### Neuromuscular data collection

#### Neuromuscular assessment

The session began with a warm-up of 10 submaximal voluntary isometric knee extensor contractions (4–5 s) between 20 and 90% of the estimated MVC. After a short recovery period, the participants performed two MVCs (4 s duration) with the right leg, separated by a 1-min rest period. For both trials, P100 Hz were delivered at maximal force, followed by a P100 Hz and a Pt in 2-s intervals.

#### Evoked contractions

A constant-current stimulator (DS7AH, Digitimer, Hertfordshire, UK) was used to deliver electrical pulses. The cathode (5 cm diameter) and the anode (5 × 10 cm, Dermatrode, American Imex Irvine, CA) were placed over the femoral nerve at the femoral triangle level below the inguinal ligament and on the lower part of the gluteal fold opposite the cathode, respectively. The optimal intensity for electrical stimulation was determined after the warm-up period by progressively increasing the stimulus intensity in 10-mA increments until there was no further increase in the amplitude of the mechanical or electrical (M-wave) responses. A 20% supplementary increment was added to ensure supramaximal stimulation intensity (Neyroud et al., 2013).

#### Force recording

Voluntary and evoked force exerted by the right knee extensors were recorded using an isometric ergometer consisting of a custom-built chair equipped with a strain gauge (Universal Load Cell, model 9363-C3, linear range 0–250 N•m, output sensitivity 2.0 mV•V^−1^, Vishay, Malvern, US). The calibrated strain gauge was fixed to the chair and strapped to the ankle with a custom-made mold. Subjects were seated with a 90-degree knee angle, the trunk was attached at a 100° angle to the chair back panel with a harness belt, and the arms had to be crossed on the chest to minimize upper body movement. The force signal was recorded at 1 kHz using an AD converter system (MP 150, BIOPAC Systems, Goleta, CA).

#### EMG recording

The EMG activity of the right vastus lateralis (VL) was recorded with a pair of silver chloride (Ag/AgCl) circular (1 cm) surface electrodes (MediTrace 100, Kendall, Canada) positioned lengthwise over the middle of the muscle belly according to SENIAM recommendations (Hermens et al., 2000); the inter-electrode distance was 2 cm. The reference electrode was placed over the patella. The VL was chosen as representative of quadriceps muscle activity (Place et al., 2007). Low resistance (<5 kΩ) was obtained by shaving, abrading and cleaning the skin. EMG signals were amplified (gain = 1000) over a frequency bandwidth of 10–500 Hz and digitized at a sampling frequency of 2 kHz using an AD converter system. Force and EMG data were analyzed offline using the software Acknowledge (Biopac System, Santa Barbara CA, USA).

### MRCP data collection

#### EEG recording

Continuous EEG was recorded at a sampling rate of 2048 Hz with a 64-channel Biosemi Active two-amplifier system (Biosemi, Amsterdam, the Netherlands) mounted according to the 10–20 International System. All channels were referenced to the CMS-DRL ground, which functioned as a feedback loop driving the average potential across the montage as close as possible to amplifier zero (Biosemi, Amsterdam, the Netherlands). Impedance was kept below 5 kΩ by using conducting gel. Participants wore the EEG cap during the entire protocol. Post-exercise recordings started 5.5 ± 0.5 min after the end of the heavy exercise and the TT, and lasted 10 min. Offline analyses were performed with BrainVision analyzer software (Brain Products Gmbh, Munich, Germany).

The MRCP data were collected using the same ergometer device used for neuromuscular assessment. To avoid any unknown disturbances induced by the neuromuscular stimulation, the other leg was used for the MRCP task. A string attached to the left ankle ran over a pulley to a free-hanging weighted platform. The subjects were instructed to lift this weight 60 times, equivalent of 20% of their MVC force, by ~10 cm. The contraction duration was not strictly controlled, but participants were instructed to produce a 2-s contraction: a 1-s concentric contraction to lift the weight, and 1-s eccentric contraction to put it down (metronome). The onset of movement was automatically reported on the EEG recording by the release of a trigger placed behind the heel of the subjects. The contractions were self-generated, but to ensure that the duration of the task was identical for each participant in each condition, a beep sounded every 10 s. The participants were instructed to perform the contraction spontaneously between two beeps. The subjects were also instructed to keep their eyes closed during the task to avoid excessive attentional load and artifacts generated by visual feedback.

### Data analysis

#### Gas exchange

Breath-by-breath pulmonary gas-exchange data were collected during the maximal ramp test with a metabolic cart (OxyconPro, Jaeger, Germany) and averaged over consecutive 10-s period. The $\overset{\cdot }{V}$*O*_2_ *peak* was taken as the highest value attained during the last 30 s before the subject's volitional exhaustion. *SV*_1_ was determined from the combination of different measurements, including the first disproportionate increase in $\overset{\cdot }{V}$*CO*_2_ from visual inspection of individual plots of $\overset{\cdot }{V}$*CO*_2_ vs $\overset{\cdot }{V}$*O*_2_, an increase in expired ventilation $\overset{\cdot }{V}$*E/*$\overset{\cdot }{V}$*O*_2_ with no increase in $\overset{\cdot }{V}$*E/*$\overset{\cdot }{V}$*CO*_2_, and an increase in end-tidal *O*_2_ tension with no fall in end-tidal *CO*_2_ tension. The intensity for the 30-min exercise was obtained by adding 20% of the difference between *SV*_1_ and MAP to the power reached at *SV*_1_.

#### Force data

MVC force of the knee extensors was reported as the force produced during the maximal voluntary contractions (i.e., peak to peak). Resting P100 Hz and Pt amplitude were analyzed for the trials yielding the highest MVC. The VAL during MVCs was estimated with the superimposed and the potentiated doublets according to the formula proposed by Strojnik and Komi (1998).

$$\begin{array}{l} VAL=(1-(superimposed\;100\;Hz\;doublet\;force\;\times \\ \;\left(force\;level\;at\;stimulation\;/MVC\;force\right)/\\ \;superimposed\;100\;Hz\;doublet\;force))\times 100 \end{array}$$

#### EMG data

The EMG signals recorded during the highest MVC were used for analysis. M-wave peak-to-peak amplitude was measured from the EMG response after the single stimulation.

#### MRCP data

The raw EEG data were first down-sampled from 2048 to 512 Hz to reduce computational load and band-pass filtered from 0.1 to 5 Hz. The low-pass filter was set at 5 Hz (Thacker et al., 2014) to avoid bias for alpha rhythm induced by the eyes-closed procedure and to avoid unwanted activity generated by spontaneous physiological and rolandic *mu* rhythms. EEG signals were segmented into 60 epochs of 3000 ms each (from 2500 ms before movement onset to 500 ms after movement onset). All trials were baseline-corrected with −2500 ms to −2000 ms as a reference and averaged using a semi-automatic artifact rejection procedure with a ±80 μV criterion. Artifacted electrodes were interpolated when necessary with a spherical 3D spline, and trials containing periods of muscular artifacts were also rejected. On average, 53 ± 9, 52 ± 11, and 52 ± 9 of 60 trials were available for analysis for the PRE, POST1, and POST2 conditions, respectively. The MRCPs were segmented into four sequential components (Shibasaki and Hallett, 2006; Jahanshahi and Hallett, 2012). The *Bereitschaftspotential* was divided into BP1 and BP2. The BP1 corresponds to the average amplitude between −1500 and −1000 ms. The BP2 corresponds to the average amplitude between −1000 and −500 ms. The third component was the negative slope (NS′), which corresponds to the average amplitude from −500 ms to the onset of movement. The last component was the motor potential (MP), taken as the maximal peak amplitude recorded between −500 ms and movement onset. Those components were calculated for two regions of interest. The first region corresponded to the mean activity of the FC1 and FC2 electrodes and the second region was the mean activity above the C2 electrode. Those electrodes were chosen because they correspond to the area known to generate MRCP, namely the SMA (represented by FC1-FC2 mean activity) for the first part of MRCP and the primary motor cortex (M1; represented by C2 activity) for the late part of MRCP (Shibasaki and Hallett, 2006).

### Statistical analysis

One-way repeated measures ANOVAs with factor Time were used to compare the neuromuscular and MRCP variables between the different times of measurement (PRE, POST1, and POST2, respectively). When ANOVA revealed significant interactions, pairwise contrasts were performed using the Bonferroni correction. Friedman ANOVAs with follow-up Wilcoxon signed rank tests were used in a few cases in which conditions for using parametric tests were not reached. To better understand the mechanisms responsible for the MVC loss, we performed Pearson correlation analyses by using the deltas between PRE-POST1 and PRE-POST2 on the factor MVC explained by VAL and P100 Hz.

Because of the non-normal distributions of EEG variables, Spearman correlations were used to explore the relationship between neuromuscular and MRCP modulations. The three neuromuscular parameters used for the correlation analyses were the reduction in MVC, VAL, and P100 Hz, which were chosen as global, central and peripheral indices of fatigue, respectively. Those parameters were correlated with the four MRCP components modulations (i.e., BP1, BP2, NS′, MP) at the FC1-FC2 and C2 electrodes. All statistical analyses were performed using the software Statistica 12.6 (Statsoft, Tulsa, USA). The level of significance was set to *p* < 0.05. The results are presented as mean ± standard deviation.

## Results

### Participants

Data from four of the original 20 recruited participants had to be excluded because of heavily artifacted EEG signals. The mean age of the 16 remaining participants was 29 ± 7 (years ± SD), and their body mass index was 22.9 ± 1.6 (kg•m^−2^). All of them were active road cyclists and/or triathlon athletes, had a total score of 9.2 ± 0.7 on the Baecke questionnaire, and reached a MAP of 385 ± 47 W at a $\overset{\cdot }{V}$*O*_2_ *peak* of 63.8 ± 5.9 ml•min^−1^•kg^−1^ during the incremental cycling test.

### Exercise data

The mean power output during the 30-min exercise was 231 ± 97 W. The mean duration for the TT was 15.7 ± 1.6 min and the average power was 279 ± 31 W. The RPE was 14.9 ± 1.7 at the end of the heavy exercise, whereas the RPE reached 19.7 ± 0.5 at the end of the TT.

### Neuromuscular data

#### Force

**Figure 2:**
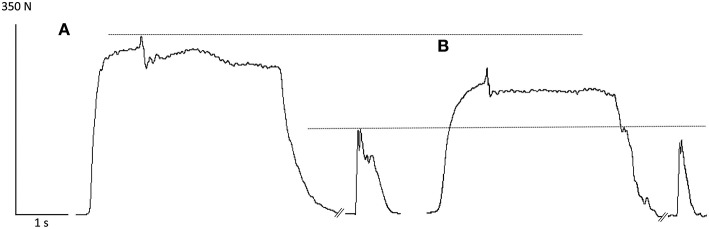
**Representative recordings of a superimposed maximal voluntary contraction with a 100 Hz-potentiated doublet in the PRE (A) and POST2 (B) conditions**. Note the decrease in MVC force and P100 Hz after the completion of the two fatiguing exercises.

The sequence of the two cycling exercises caused a significant reduction in the MVC force measured at POST1 and POST2 [*F*_(2, 30)_ = 55.34, *p* < 0.001; Table 1]. Figure 2 shows two representative recordings of a superimposed MVC with a 100 Hz-potentiated doublet in the PRE (A) and POST2 (B) conditions.

**Table 1:** **Neuromuscular indices of central and peripheral fatigue measured before the fatiguing task (PRE), after the heavy exercise (POST1), and after the 10-km time trial (POST2)**.

	**PRE**	**POST1**		**POST2**	
	***Mean* /± SD**	***Mean* /± SD**	**/Δ PRE % /± SD**	***Mean* /± SD**	**/Δ PRE % /± SD**
MVC (N)	306± 54	276± 55^**^	−10± 8	244± 51^**^	−21± 9
VAL (%)	90.9± 5.1	85.6± 9.5^*^	−5.9± 8.4	80.5± 11.5^**^	−11.6± 10.4
Pt (N)	93.3± 21.3	72.7± 19.4^**^	−20.9± 15.8	61.2± 14.2^**^	−32.4± 16.7
P100 Hz (N)	132.7± 30.1	122.6± 28.8^**^	−6.9± 10.69	116.5± 30.9^**^	−12.1± 13.2
M-wave (mV)	4.8± 2.3	4.6± 2.3	−3.9± 15.2	4.3± 2.3^**^	−10± 15.5

MVC, maximal voluntary contraction; VAL, voluntary activation level with reference to the 100 Hz resting peak doublet. Pt, muscle twitch; P100 Hz, resting peak doublet at 100 Hz; M-wave, peak to peak M-wave amplitude. Δ PRE, percentage of difference from PRE. Significant differences from PRE:

* p < 0.05;

** *p < 0.01*.

#### Peripheral and central fatigue

The peripheral indices of fatigue measured by the Pt force and P100 Hz were significantly reduced [*F*_(2, 30)_ = 36.95, *p* < 0.001 and Chi^2^ (*N* = 16, df = 2) = 6.5, *p* = 0.039], respectively after POST1 and POST2 (Table 1). For the M-wave amplitude, Friedman ANOVA revealed a significant effect of Time [Chi^2^ (*N* = 16, df = 2) = 9.5, *p* = 0.008]. However, although no reduction was observed between PRE and POST1 (*p* = 0.3), the difference in the M-wave amplitude between PRE and POST2 was significant (*p* = 0.007). Concerning central fatigue, a Time effect [*F*_(2, 30)_ = 14.21, *p* < 0.001] was observed for VAL. For all the significant main Time effects, *post-hoc* tests revealed a significant difference between PRE and POST1 (except for M-wave) and between PRE and POST2 (All *p* < 0.033; Table 1).

#### Correlations

At POST1, Pearson correlation analysis showed a trend toward a relationship between the reduction in MVC force and the reduction in P100 Hz (*r* = 0.44, *p* = 0.08), but without reaching the level of significance. No relationship was found between MVC and VAL reduction (*r* = 0.32, *p* = 0.22). At POST2, the results indicated no significant relationship between the decrease in MVC and P100 Hz (*r* = 0.27, *p* = 0.3), whereas a positive relationship was found between the reduction in MVC and VAL (*r* = 0.5, *p* = 0.047).

### MRCP data

**Figure 3:**
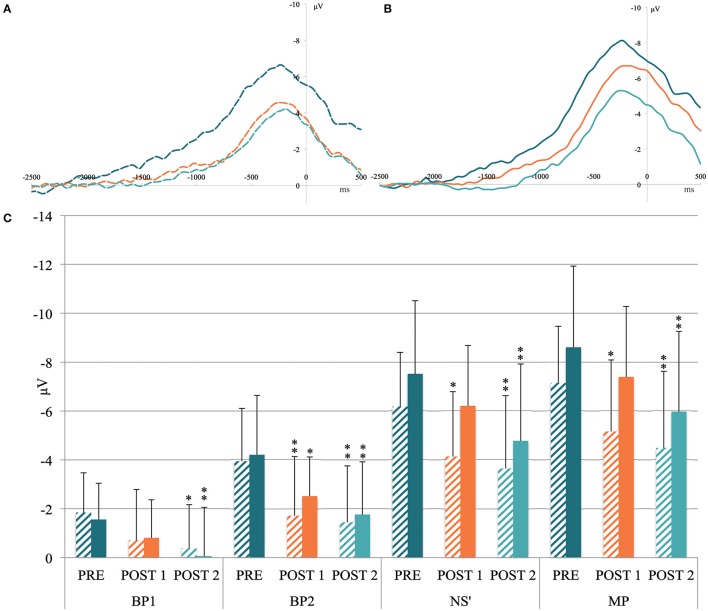
**(A)** Grand MRCP average recorded above the FC1-FC2 (i.e., above supplementary motor area) and **(B)** C2 electrodes (i.e., above primary motor cortex) in the pre-exercise condition (dark blue curve), after the heavy exercise (orange curve) and after the 10-km time trial (light blue curve). X-axis units: time in milliseconds locked to the movement onset. Y-axis units: amplitude in microvolts. **(C)** Mean activity of movement-related cortical potential components measured before the cycling task (PRE), after the heavy exercise (POST1), and after the 10-km time trial (POST2) on the FC1-FC2 electrodes (shaded column) and the C2 electrode (filled column). BP1, mean activity from −1500 to −1000 ms; BP2, mean activity from −1000 to −500 ms; NS', mean activity from −500 ms to movement onset; MP, peak amplitude. Significant differences from PRE: ^*^p < 0.05; ^**^p < 0.01.

The MRCP grand averages between the PRE, POST1, and POST2 conditions at FC1-FC2 and C2 are shown in Figures 3A,B. The common MRCP shape can be observed, with a typical increase in slope at ~1000 and 500 ms before movement onset.

#### Mean activity at FC1-FC2

ANOVA revealed an effect of Time for FC1-FC2 mean activity for the components BP1 [*F*_(2, 30)_ = 3.93, *p* = 0.03], BP2 [*F*_(2, 30)_ = 7.73, *p* = 0.019], NS′ [*F*_(2, 30)_ = 7.17, *p* = 0.003], and MP [*F*_(2, 30)_ = 7.39, *p* = 0.002; Figure 3C]. The *post-hoc* tests indicated a significant reduction between PRE and POST1 for BP2 (*p* = 0.007), NS′ (*p* = 0.012), and MP (*p* = 0.016). At POST2, the reduction was significant for the four MRCP components (All *p* < 0.04; Figure 3C).

#### Mean activity at C2

ANOVA revealed a Time effect on the C2 mean activity for the component BP1 [*F*_(2, 30)_ = 4.91, *p* = 0.014], BP2 [*F*_(2, 30)_ = 8.83, *p* < 0.001], NS′ [*F*_(2, 30)_ = 9.14, *p* < 0.001], and MP [*F*_(2, 30)_ = 8.43, *p* = 0.001]. The *post-hoc* tests indicated a significant decrease between PRE and POST1 only for B2 (*p* = 0.01), whereas the reduction was significant for the four components between PRE and POST2 (all *p* < 0.012; Figure 3C).

### Neuromuscular and MRCP correlations

Bonferroni *post-hoc* tests revealed significant changes for the MRCP components between PRE-POST1 and between PRE-POST2 conditions, whereas no changes were observed between POST1 and POST2 (see Figure 3). Therefore, the correlation analyses between MRCP and neuromuscular modulations were based solely on the PRE-POST1 and PRE-POST2 differences.

**Figure 4:**
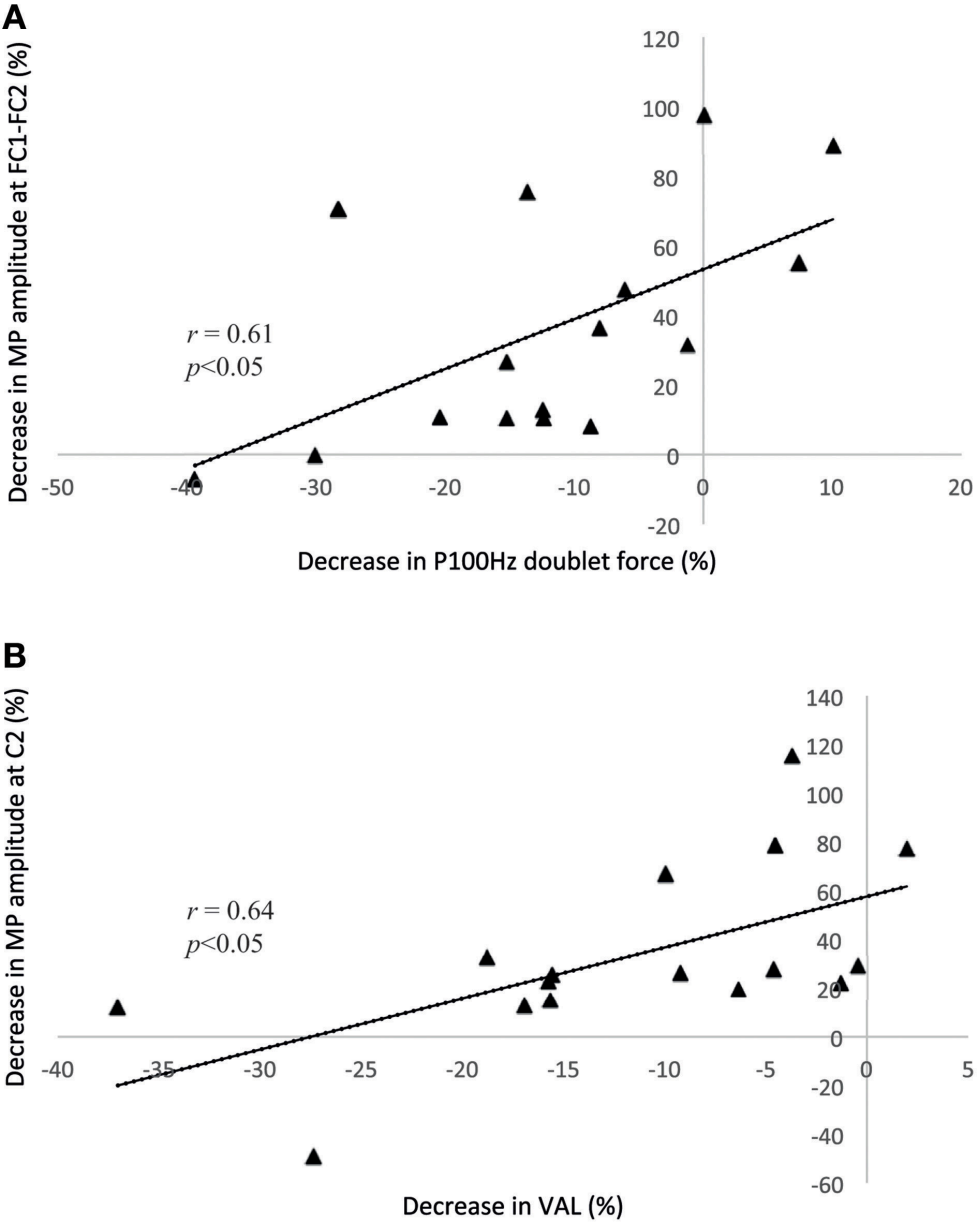
**Correlation analyses between the P100 Hz doublet force and the motor potential above the C2 electrode (A) and between the voluntary activation level and the decrease in the motor potential above the FC1-FC2 electrodes (B)**. Both correlations are based on the differences between the PRE and POST2 conditions. Note that the pre-motor potential is a negative value, and thus, a reduction in the amplitude represents a change toward zero (i.e., the value becomes less negative). MP, motor potential; VAL, voluntary activation level.

No correlation was found between PRE and POST1. In contrast, between PRE and POST2, the reduction in P100 Hz was correlated with the decrease in NS′ (*r* = 0.61) and the MP (*r* = 0.61) amplitude above FC1-FC2 (all *p* < 0.05), whereas the reduction in VAL was correlated with the decrease in BP1 (*r* = 0.57), BP2 (*r* = 0.65), NS′ (*r* = 0.72), and MP (*r* = 0.64) above C2 (all *p* < 0.05). The correlations between the reduction in VAL and MP above FC1-FC2 and between the reduction in P100 Hz and MP amplitude above C2 are illustrated in Figure 4. Note that the pre-motor potential is a negative value, and thus, a reduction in the amplitude represents a change toward zero (i.e., the value becomes less negative).

## Discussion

The current study was designed to induce neuromuscular fatigue by using two successive cycling exercises, at heavy and severe intensities, and to assess the related effects on MRCP. The second aim was to relate the exercise-induced MRCP modulations with neuromuscular alterations. Although the large-muscle-group exercise induced neuromuscular fatigue, the results indicated a reduction in MRCP amplitude instead of an increase as expected. The cycling exercise induced both peripheral alterations (as indicated by the decreased P100 Hz and Pt) and central impairments (as indicated by the reduction in VAL). The exercise intensity difference between the heavy exercise and the TT was indicated by a higher perception of effort and a greater strength loss after the second exercise. After heavy exercise, MRCP was reduced mainly above the FC1-FC2 electrodes, whereas the MRCP reduction observed at the end of the TT was associated with the FC1-FC2 and C2 electrodes. The relationship found between the reduction in the late MRCP components (i.e., NS′, MP) and P100 Hz above FC1-FC2 and with VAL above C2 indicated a close interaction between neuromuscular fatigue and pre-motor brain activity.

### Physical exercise and neuromuscular fatigue

An MVC loss of 10% was observed after the heavy exercise. As expected, this reduction was associated with peripheral alterations characterized by a decrease of 20% in Pt force, without changes in the VL M-wave amplitude and with central fatigue, as reflected by the decrease in VAL. Such neuromuscular changes are very similar to those reported by Lepers et al. (2001). After a cycling exercise lasting 30 min at 80% MAP among trained athletes, the authors reported a decrease of 13% in knee extension force, accompanied by a reduction of 20% in the Pt without changes in the M-wave properties, suggesting alteration of processes located beyond action potential propagation/transmission. Such a reduction in Pt force may be related to intracellular disturbances, such as reduced Ca^2+^ release from the sarcoplasmic reticulum, decreased sensitivity of myofilaments to Ca^2+^, changes in metabolite (H^+^, inorganic phosphate) concentrations within the muscle, and/or reduced force produced by each active cross-bridge (Allen et al., 2008). The absence of significant correlations between MVC force loss and central or peripheral markers of fatigue between PRE and POST1 does not allow for a clear determination of the origin of fatigue. However, relying on other studies demonstrating that peripheral fatigue develops early during such exercise (Decorte et al., 2010), we believe that the trend observed between the MVC force loss and the reduction in P100 Hz in POST1 favors a major role for peripheral alterations.

After the severe intensity exercise, knee extensor MVC force was decreased by 21%. This strength loss is comparable to the results of Lepers et al. (2001), who reported a reduction of 16% after 30 min of cycling at 80% of PMA. As at POST1, peripheral and central alterations participated in knee extensor force impairment at POST2. The additional peripheral fatigue (−32% in Pt at POST2 vs. −21% at POST2) can be attributed to alteration in action potential transmission/propagation, as indirectly indicated by the 10% reduction in VL M-wave amplitude at POST2. The finding that the VL M-wave amplitude decreased at POST2 but not at POST1 confirms the results of Lepers et al. (2002, 2004) suggesting that cycling exercise must be of sufficient intensity and duration to affect muscle excitability. The purpose of performing the 10-km time trial after having previously performed a heavy exercise lasting 30 min at 60% of MAP (i.e., 67% $\overset{\cdot }{V}$*O*_2_ *peak*), with only a 15-min break (i.e., for data collection), was also to generate greater central fatigue. Indeed, VAL was 91% in PRE condition and decreased to 80% at POST2, in accord with the literature (Lepers et al., 2001; Millet and Lepers, 2004). This finding suggests altered CNS functioning leading to a limitation of descending motor drive. Overall, our results indicate that peripheral mechanisms were mainly involved in the development of fatigue at POST1, whereas central fatigue played a major role in force reduction at POST2.

### MRCP data

Before, between and after exercise, we observed MRCPs with a shape and amplitude similar to those reported in the literature (Shibasaki and Hallett, 2006). The MRCPs were characterized by a slow negative shift starting between 1500 and 2000 ms before movement onset, with a typical change in slope occurring at approximately −500 ms (Figure 3). Our data indicate that this inflection occurred slightly earlier (around −750 ms), likely because the trigger was set at the onset of movement instead of the onset of EMG activity. Both fatiguing cycling tasks resulted in a decrease in MRCP amplitude recorded during 60 spontaneous knee extensions at 20% of MVC. A long-term effect on brain activity was already reported by Thacker et al. (2014), who showed MRCP modulations 1 h after the end of an endurance exercise. Our results extend these findings by showing that the large-muscle-group exercise led to distinctive changes in the pre-motor potential during the two 15-min periods post-exercise (POST1 and 2) compared with the fresh condition (PRE).

The present findings do not confirm that pre-motor brain activity increases with muscle fatigue, as expected and as previously reported in repeated single-joint contraction protocols. Our study used a dynamic large-muscle-group-fatiguing task quite different from the task used to quantify changes in MRCP. It is therefore difficult to compare our results with those of previous single-joint contractions protocols. Several authors have asked their experimental subjects to perform repetitive blocks of contractions and have compared pre-motor potential amplitudes between the first and the last blocks. In such designs, fatigue is not experimentally manipulated through a specific fatiguing task. Another difference from our design is the potential for mental load. In most studies, participants have had to achieve a given force level during contraction by using visual feedback. A high degree of concentration must be maintained to correctly perform the task throughout the block of repeated contractions. However, it has been suggested that an increase in attentional load and the mobilization of cognitive resources could be a confounding factor resulting in an increase in MRCP amplitude (Freude and Ullsperger, 1987; Berchicci et al., 2013). Another factor to consider is the contraction force level used during the MRCP task. In the studies of Johnston et al. (2001) and Schillings et al. (2006), the MRCP task consisted of contractions at 70% of the MVC. By comparing the premotor potential amplitude between the first and last blocks of contractions, the authors reported an increase in MRCP. According to the authors, this result suggested that the cortical activity compensated for the reduction in strength capacities (i.e., to provide the same level of force, the brain had to mobilize more resources). In the present study, the load lifted during the MRCP task was kept low (20% of MVC force) and additional cortical activation to compensate for peripheral fatigue did not appear to be required. In a similar vein, Morree et al. (2012) did not observe any increase in pre-motor potential after repeated contractions at 20% of MVC force despite a final maximal force loss of 35%.

The difference in the effects on pre-motor potential observed in this study and others (i.e., a reduction in pre-motor vs. an increase, respectively) may arise from the differing fatiguing task features. The effect of single-joint contraction tasks on motor cortex excitability measured by transcranial magnetic stimulation has been reported to be different from the effect observed for locomotor exercises. The excitability of the motor cortex increases after a fatiguing single-joint contraction task, but after a 30-min steady-state sustained cycling exercise, Sidhu et al. (2012) reported no increase in the responsiveness of the motor cortex. Modulation of cortical excitability is likely task-specific and may be related to the systemic physiological consequences of large-muscle-group exercise. The reduction in MRCP could be related to input from group III and IV muscle afferents to the brain. Indeed, fatiguing voluntary contraction lengthens the cortical silent period, as measured by transcranial magnetic stimulation, which is believed to reflect intracortical inhibitory activity (Gruet et al., 2013). However, when the activation of group III and IV muscle afferents are artificially blocked with an anesthetic solution (fentanyl) injection, the cortical silent period is not prolonged, indicating a modulation of intracortical inhibitions (Hilty et al., 2011b). Similarly, maintaining muscle afferent activity at the end of a fatiguing task with ischemia reduces motor cortex output that maximally activates the muscle (Gandevia et al., 1996). More generally, endurance exercise modulates several additional parameters that might play a role in the reduction of the pre-motor potential, such as cerebral oxygenation (Ide and Secher, 2000), brain catecholamines (Nybo and Secher, 2004), or hyperthermia (Périard et al., 2011).

To the best of our knowledge, only one study has investigated MRCP modulations after a large-muscle-group endurance exercise (Thacker et al., 2014). The exercise consisted in pedaling for 20 min at 70% of the age-predicted maximum heart rate on a cycle ergometer. To remove confounding and fatigue factors, the authors used an MRCP task involving the upper limbs (i.e., wrist extension). The results indicated no changes in MRCP amplitude or onset immediately after the end of exercise. We assume that the absence of modification was caused by insufficient exercise intensity or durations and/or because the muscle group used for the MRCP task was different from that mobilized for the fatiguing task.

In our protocol, a reduction in MRCP was observed during movement preparation, initiation, and movement onset, as reflected by the reduction in the BP, NS′, and MP components, respectively. The heavy exercise induced a decrease in MRCP amplitude for the components BP2, NS′, and MP above FC1-FC2, whereas only BP2 decreased significantly above C2. After the TT, all MRCP components decreased significantly from the PRE condition above the FC1-FC2 and C2 electrodes. Our results indicate that heavy exercise affects the MRCP differently if it is recorded above SMA or M1. The SMA is connected to the subcortical region, relays sensory feedback received from the muscle and sends direct signals to the M1 (Dum and Strick, 1991; Cadoret and Smith, 1997; Colebatch, 2007), such that the neurons in the SMA are activated several milliseconds before activity within the pyramidal tract (Eccles, 1982). Thus, it appears that the modulations observed above the SMA are more closely related to the integration of peripheral mechanisms, whereas the modulations above the primary motor cortex are more likely to reflect the cortico-motor command. The positive correlation between the increase in peripheral fatigue measured by P100 Hz and the reduction in MRCP (i.e., NS′ and MP) above the SMA strengthens the assumption that this area may be under the modulatory influence of muscle activity, likely via type III and IV afferents. This assumption is also supported by the studies of Gandevia (2001) and Amann et al. (2011) which showed that type III/IV muscle afferents are likely to exert an inhibitory effect on central motor drive during whole-body exercise.

At POST1, the components related to movement preparation are first altered without affecting the execution period of movement production, as reflected by no differences in NS′ and MP amplitude above M1. When neuromuscular fatigue is more pronounced, as observed at POST2, the components related to movement preparation and execution are both modulated, showing a growing impact of fatigue on all the components involved in movement production. The activation of the corticospinal tract via the primary motor cortex is the final step of movement production. Thus, when exercise-induced fatigue decreases the MP above the SMA and M1, it is possible that the motor cortex does not reach the level of activity required to produce the same level of voluntary activation, resulting in a greater decrease in MVC force, as observed at POST2. The positive correlation between the decrease in VAL and the reduction in MRCP above M1 support this assumption, as well as the positive correlation between the drop in MP amplitude above M1 and the reduction in MVC force. Interestingly, some authors have reported neural plasticity between the SMA and M1. By using transcranial magnetic stimulation, Arai et al. (2012) showed that the motor-evoked potential generated by stimulating M1 can be modulated by an SMA-conditioning stimulation procedure. Recently, Bajaj et al. (2015) also showed that the neural connectivity between the SMA and M1 could be modulated by therapy in stroke survivors. It is not unlikely that the acute fatiguing task performed in our study reorganized the connectivity between the SMA and M1 and that the neural impulses sent from SMA to M1 were reduced under the influence of afferent projections.

The limits of our study concern the factors related to movement characteristics, such as strength, accuracy, or rate of force development, all of which are known to modulate the MRCP (Shibasaki and Hallett, 2006). In our protocol, the range of motion was the same between trials. However, the movement duration was not controlled, and we cannot exclude that the duration between the onset of muscle activity and the onset of movement did not change with fatigue. In addition, the MRCP modulations reported in this study are specific to well-trained athletes and cannot be extended to the general population. Because neuroelectric activity could be related to physical fitness (Kamijo et al., 2010), further studies have to be repeated in different populations to determine the impact of fitness on MRCP, especially after endurance exercises.

In conclusion, the MRCP reflects the intention to move and the preparatory period for the intended movement. The results of our study indicate that a cycling exercise induces peripheral and central fatigue and reduces MRCP amplitude. The MRCP components related to movement planning and initiation above the SMA area first altered by heavy exercise-related fatigue, likely by peripheral muscle activity. When neuromuscular fatigue is substantial, as observed after the TT, the overall reduction in MRCP, especially the reduction in the brain component related to movement execution above the primary motor cortex (i.e., MP), is associated with the reduction in the maximal voluntary level, resulting in a decrease in maximal voluntary force.

Finally, large-muscle-group exercise induces neuromuscular fatigue, resulting in an alteration of the corticospinal command. Because this study indicates that this command is modulated by pre-motor cortical activity, we now suggest taking a step backwards to investigate post-exercise resting state electro-cortical dynamics, from which the intention to move emerges.

## Author contributions

JNS, FB, JB: Contributions to the conception of the work. JNS, FB, NP, BK, JB: Contributions the acquisition and interpretation of data. JNS, FB, NP, BK, JB: Revising the content. JNS, FB, NP, BK, JB: Final approval of the version.

### Conflict of interest statement

The authors declare that the research was conducted in the absence of any commercial or financial relationships that could be construed as a potential conflict of interest.
